# Hyperpolarization-Activated Current (*I*_h_) in Ganglion-Cell Photoreceptors

**DOI:** 10.1371/journal.pone.0015344

**Published:** 2010-12-20

**Authors:** Matthew J. Van Hook, David M. Berson

**Affiliations:** Department of Neuroscience, Brown University, Providence, Rhode Island, United States of America; Oregon Health and Science University, United States of America

## Abstract

Intrinsically photosensitive retinal ganglion cells (ipRGCs) express the photopigment melanopsin and serve as the primary retinal drivers of non-image-forming visual functions such as circadian photoentrainment, the pupillary light reflex, and suppression of melatonin production in the pineal. Past electrophysiological studies of these cells have focused on their intrinsic photosensitivity and synaptic inputs. Much less is known about their voltage-gated channels and how these might shape their output to non-image-forming visual centers. Here, we show that rat ipRGCs retrolabeled from the suprachiasmatic nucleus (SCN) express a hyperpolarization-activated inwardly-rectifying current (*I*_h_). This current is blocked by the known *I*_h_ blockers ZD7288 and extracellular cesium. As in other systems, including other retinal ganglion cells, *I*_h_ in ipRGCs is characterized by slow kinetics and a slightly greater permeability for K^+^ than for Na^+^. Unlike in other systems, however, *I*_h_ in ipRGCs apparently does not actively contribute to resting membrane potential. We also explore non-specific effects of the common *I*_h_ blocker ZD7288 on rebound depolarization and evoked spiking and discuss possible functional roles of *I*_h_ in non-image-forming vision. This study is the first to characterize *I*_h_ in a well-defined population of retinal ganglion cells, namely SCN-projecting ipRGCs.

## Introduction

Intrinsically photosensitive retinal ganglion cells (ipRGCs) are a unique class of retinal output neurons distinguished by their expression of the photopigment melanopsin and their direct sensitivity to light [1], [2]. They serve as the dominant or sole source of direct retinal influence on non-image-forming visual processes such as circadian photoentrainment, the pupillary light reflex, and suppression of melatonin production in the pineal [3]–[5]. Thus far, several types of ipRGCs, termed M1 through M5, have been described in the literature [6]–[10]. M1 cells are the major source of retinal input to the suprachiasmatic nucleus of the hypothalamus (SCN), the central circadian pacemaker [7]. Their dendrites stratify in outermost sublamina of the inner plexiform layer (IPL). Other ipRGC classes stratify in the inner layers of the IPL [8]–[11] and innervate an array of central targets including the superior colliculus, the olivary pretectal nucleus, posterior pretectal nucleus, and lateral geniculate nucleus [7], [10].

Photoactivation of melanopsin triggers a phosphoinositide signaling cascade closely resembling that of invertebrate rhabdomeric photoreceptors [12]–[17]. This gates an inward current that depolarizes ipRGCs and triggers action potentials. Additionally, like other RGCs, ipRGCs receive rod and cone-driven synaptic inputs that influence their net output to central targets [18]–[20].

While several studies have examined the intracellular cascades and ion channels involved in these intrinsic and synaptically driven light responses [14], [16], [17], [19]–[21], little is known about the voltage-gated currents expressed by ipRGCs.

In surveying published evidence relevant to this issue, we were struck by some data illustrated by Hartwick et al. [16] in their study of dissociated ipRGCs. Hyperpolarizations produced by prolonged current steps did not reach a steady plateau, but rather drifted gradually back toward the resting potential. This depolarizing voltage rectification, also called a depolarizing voltage “sag,” is characteristic of cells possessing the hyperpolarization-activated cation current *I*_h_, and has been described in other studies of retinal ganglion cells [22]–[26]. *I*_h_ is characterized by a slowly-developing inward cation current triggered by membrane hyperpolarization and is carried by Na^+^ and K^+^ with a slight preference for the latter ion [27]. It is also known as *I*_f_ (for “funny” current) in the sinoatrial node of the heart, where it underlies the heart's pacemaker activity. *I*_h_ is carried by four subtypes of HCN channels (for “hyperpolarization-activated, cyclic nucleotide-gated”), HCN1-HCN4 [28], [29].

Though *I*_h_ is expressed in retinal ganglion cells, the level of expression apparently varies among RGC types. This is most clearly established in cat retina [24], but a study of rat ganglion cells suggests a similar pattern [26]. There has been no prior study specifically linking *I*_h_ to ipRGCs in any species. Here we use electrophysiological techniques to confirm the presence of *I*_h_ in rat ipRGCs and to characterize this current in detail. We find that its characteristics are consistent with those of *I*_h_ in other systems, including rat RGCs. We show that *I*_h_ is not involved in setting the ipRGC resting membrane potential and discuss its possible roles in the non-image-forming light responses of ipRGCs.

## Materials and Methods

### Retrograde labeling and retinal dissociation

All animal procedures were conducted in accordance with National Institutes of Health guidelines and approved by Brown University's Institutional Animal Care and Use Committee (protocol # 0905050). The Animal Welfare Assurance Number for Brown University is A3284-01. Adult male Sprague-Dawley rats (240–300 g) were housed in a 12∶12 hour light∶dark cycle with food and water provided *ad libitum*.

ipRGCs were labeled by retrograde transport of rhodamine-labeled latex microspheres from the SCN, as described previously [17], [30]. At least 48 hours after the labeling surgery, animals were sacrificed by CO_2_ asphyxiation and their retinae screened for labeling by epifluorescence. Labeled retinae were digested in a solution of papain following the methods of Meyer-Franke et al. [31] and Graham et al. [17] and incubated overnight on coverslips coated with laminin and poly-D-lysine in a culture medium of Neurobasal-A (Invitrogen; Carlsbad, CA) supplemented with L-glutamine (1 mM; Invitrogen), ciliary neurotrophic factor (10 ng/ml; Sigma; St. Louis, MO), brain-derived neurotrophic factor (25 ng/ml; Sigma), forskolin (5 µM; Tocris; Ellisville, MO), B-27 (1x; Invitrogen) and gentamycin (10 µg/ml; Invitrogen).

### Electrophysiological recording

Individual coverslips were mounted with vacuum grease onto a ∼3 ml recording chamber and superfused constantly with Ames' medium (Sigma; 2–4 ml/min, 30–33°C) that contained (in mM) 120 NaCl, 3.1 KCl, 1.15 CaCl_2_, 1.24 MgSO_4_, 0.52 KH_2_PO_4_. The Ames' medium was supplemented with 10 mM D-glucose and 23 mM NaHCO_3_ and bubbled with 5% CO_2_ in O_2_. To ensure that components of the culture medium were thoroughly washed away during recording, all cells were superfused with Ames' medium for >30 minutes, and many for >1 hour, before whole-cell recording.

Pipettes were pulled from thick walled borosilicate tubing with a Flaming/Brown P-97 pipette puller (Sutter Instruments; Novato, CA) and had tip resistances of 4–9 MΩ when filled with a standard internal solution containing (in mM) 120 K-gluconate, 5 NaCl, 4 KCl, 2 EGTA, 10 HEPES, 4 ATP-Mg, 7 phosphocreatine-Tris, and 0.3 GTP-Tris (260–280 mOsm). The pH was adjusted to 7.3 with KOH. For experiments conducted in perforated patch configuration, we included amphotericin-B (200 µM) in the standard pipette solution.

Labeled cells were identified by epifluorescence using an upright microscope equipped with a 40× water immersion lens, then given 15–30 mins to dark-adapt, after which they were viewed only under infrared illumination. Cells were targeted for voltage-clamp or current-clamp recordings in either the whole-cell or perforated-patch configuration. In perforated patch experiments, we waited 15–45 mins after establishing a giga-ohm seal before beginning an experiment to allow the series resistance to fall below 50 MΩ. Series resistance was not compensated in voltage-clamp recordings and whole-cell experiments were discarded if the series resistance exceeded 30 MΩ. Isolated cells were voltage clamped at ^−^73 mV after correction for liquid junction potential (calculated to be ^−^13 mV for the standard internal solution). For current-clamp recordings, we compensated for series resistance errors during current injections by calculating the voltage drop across the series resistor and subtracting that value from the recorded voltage response.

For photic stimulation, isolated cells were illuminated from below through the microscope's condenser lens with unfiltered, broad-band light from a 100 W tungsten-halogen lamp. Stimulus irradiance (in photons×s^−1^×cm^−2^) was 2.8×10^14^ at 480 nm.

Recordings were made with a Multiclamp 700A amplifier (Axon Instruments/Molecular Devices; Sunnyvale, CA). Signals were low-pass filtered at 4 kHz and sampled at 10–20 kHz. pClamp 9 (Axon Instruments/Molecular Devices) was used for data acquisition.

CsCl was added directly to Ames' medium while ZD7288 (4-ethylphenylamino-1,2-dimethyl-6-methylaminopyrimidinium chloride; Tocris; Ellisville, MO) was dissolved in H_2_O to make a stock solution which was then diluted in Ames' medium.

### Data analysis

Traces were further low-pass filtered offline from 50–300 Hz for analysis and display. pClamp 10, Microsoft Excel, and Origin 6 (OriginLab; Northampton, MA) were used for data analysis.

To measure time constants of activation and deactivation, traces were fit with a single exponential in pClamp.

To calculate the relative Na∶K permeability, we used a tail current analysis. The equilibrium potential (E_rev_) of the fully-activated *I*_h_ was determined by linear extrapolation of a plot of tail current amplitude as a function of holding potential. We assumed that Na^+^ and K^+^ were the only ions contributing to this current. E_rev_ for each cell was used in the Goldman-Hodgkin-Katz equation:
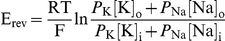
where T is the absolute temperature, R is the gas constant, and F is Faraday's constant. *P*_K_, which is the permeability to K^+^, was set at 1 so we could solve for the relative permeability to Na^+^, *P*_Na_.

Activation curves were constructed from ZD7288 or Cs^+^-sensitive tail currents fit with a single exponential to extrapolate back to the point of repolarization from various hyperpolarized test potentials. The tail current amplitudes were normalized to the tail current from the ^−^123 mV test potential and were fit with a Boltzmann equation:
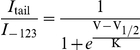
where V is the test potential, V_1/2_ is the half-maximal voltage of activation, *I*_tail_/*I*_−123_ is the normalized tail current amplitude, and K is the slope factor.

Threshold for *I*_h_ activation was determined as the membrane potential (V) at 5% of maximal activation (*I*_tail_/*I*_−123_ = 0.05).

Input resistance (R_N_) was calculated as the slope of a linear fit of the steady-state voltage deflection evoked by a series of hyperpolarizing current injections from ^−^60 to 0 pA. Any small changes in membrane potential after application of pharmacological agents were corrected by DC injection before subsequent measurements.

We used the Event Detection function in pClamp to count the number of action potentials evoked by current injection or a light flash. All events were also inspected by eye. For measuring light-evoked spiking, we counted the number of action potentials from the onset of the 1-second light stimulus to the end of the 20-second recording. Cells that entered depolarization block following light stimulation were excluded from this analysis.

Unless otherwise noted, data are expressed as mean ± SD and data were considered significant when *P*<0.05 as determined using a dependent two-tailed Student's *t*-test.

## Results

### *I*_h_ in ipRGCs

**Figure 1:**
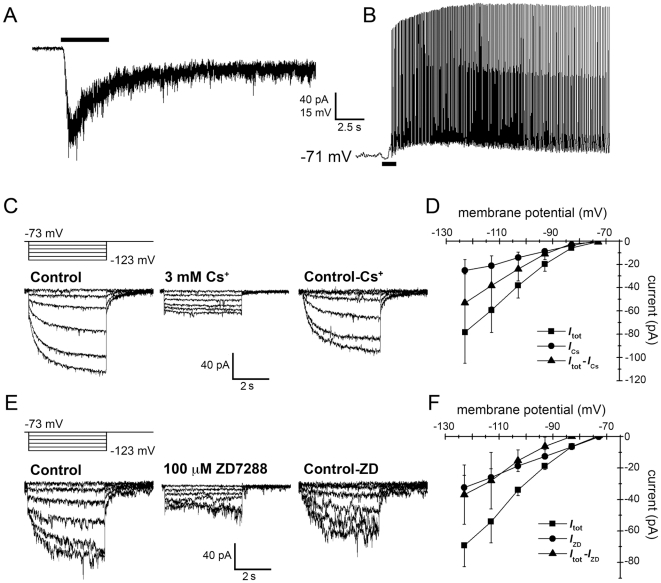
Light responses in isolated ipRGCs and evidence for expression of the hyperpolarization-activated inwardly-rectifying current (*I*_h_). **A**, **B**) Light-evoked responses recorded from dissociated ipRGCs, retrolabeled by injection of rhodamine labeled latex microspheres into the suprachiasmatic nucleus. **A**) Voltage clamp recording of a typical ipRGC. Cell was held at ^−^73 mV and given a 4 s flash of white light (black bar), triggering a large inward current. **B**) Current clamp recording from a different ipRGC. A 1 s light flash (black bar) depolarized the cell, causing spiking that persisted several minutes after termination of the light stimulus. **C–F**) Evidence for the presence of *I*_h_. **C**) Hyperpolarizing the membrane in 4 s steps evoked an instantaneous current response that, at membrane potentials negative to ^−^83 mV, was followed by a slowly activating inward current. Bath-application of 3 mM Cs^+^ to block *I*_h_ abolished the slow component. **D**) Group data (*N* = 6) plotting the total current (squares; *I*_tot_), the Cs^+^-insensitive current (circles; *I*_Cs_), and the difference between them (triangles; *I*_tot_-*I*_Cs_), which isolates the Cs^+^-sensitive, presumptive *I*_h_ current. **E**, **F**) Bath-application of the alternative *I*_h_ blocker 100 µM ZD7288 likewise abolished the slow component (*N* = 4).

We recorded from a total of 121 isolated ipRGCs. Each of these cells exhibited a direct photoresponse, consisting of an inward current in voltage-clamp recordings (Figure 1A; average amplitude 62±39 pA) and a depolarization and train of action potentials in current clamp (Figure 1B). Both the current and voltage responses to light exhibited the slow post-stimulus decay typical of melanopsin-mediated photoresponses.

**Figure 2:**
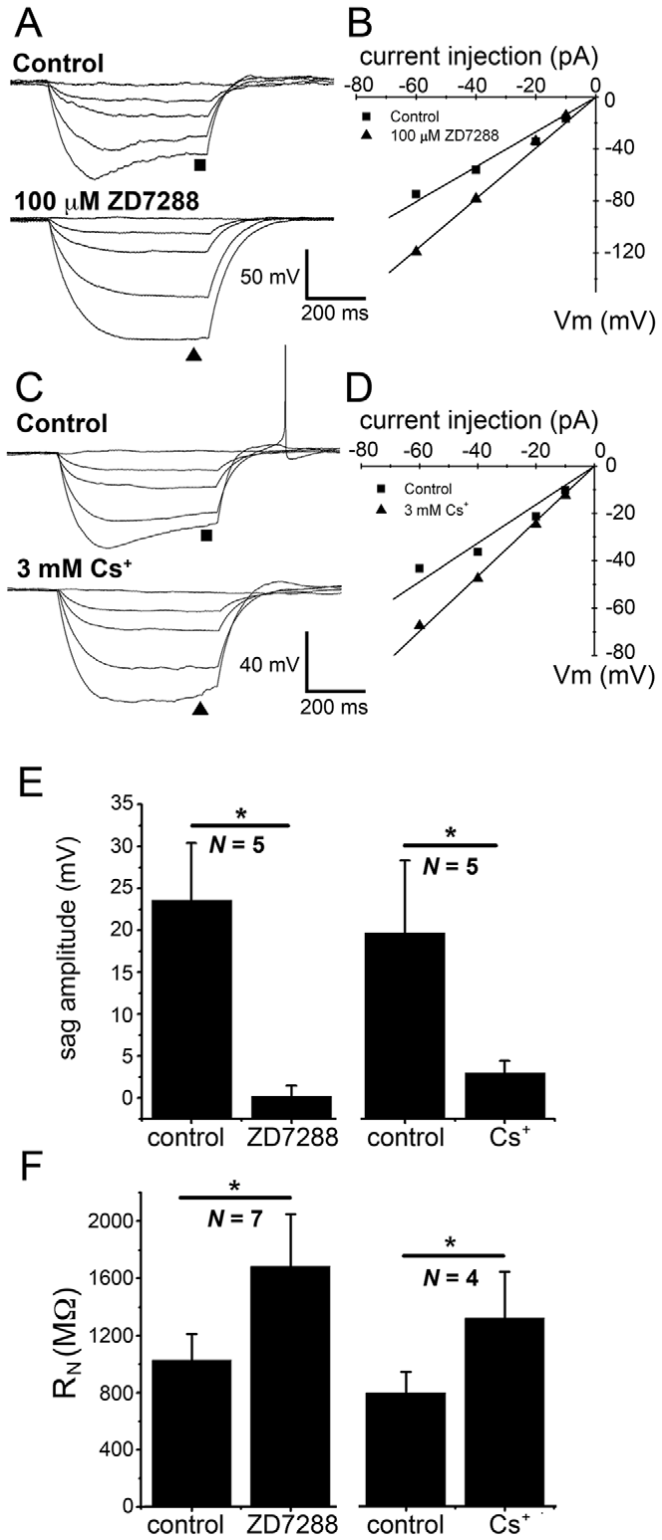
The depolarizing sag in ipRGCs and its mediation by *I*_h_. **A**–**D**) Analysis of the hyperpolarizations evoked in ipRGCs by current injections of 0, ^−^10, ^−^20, ^−^40 and ^−^60 pA (500 ms). For larger current steps, membrane potential sagged from its peak hyperpolarization back toward resting potential (Panels **A** and **C**; ‘Control’). **A**) Depolarizing sag in an example cell (top) and its blockade (bottom) by bath application of the *I*_h_ blocker ZD7288 (100 µM). **B**) Current-voltage plots of data in **A** drawn from the end of the current pulse, at the time marked by symbols in **A** (squares: control conditions; triangles: in presence of drug). Input resistance (R_N_), calculated as the slope of the linear fit to these current-voltage data, was increased by the *I*_h_ antagonist. **C**, **D**) Extracellular Cs^+^ (3 mM), an alternative *I*_h_ blocker, reproduced the effects of ZD7288 in another ipRGC, blocking the depolarizing sag (**C**) and increasing R_N_ (**D**). Resting potential was maintained at ^−^72 mV in both this cell and that in **A** (see methods). **E**, **F**) Group data showing that both ZD7288 and Cs^+^ dramatically attenuate the depolarizing sag (**E**; measured as the peak-to-steady state potential difference during ^−^60 pA current injections) and increase R_N_ (**F**; measured as in **B** and **D**). * *P*<0.05.

Nearly all of these ipRGCs (94%; 114/121) showed electrophysiological responses characteristic of *I*_h_. In voltage clamp recordings, this consisted of a slowly developing inward current triggered by membrane hyperpolarization (Figure 1C–F). In current-clamp recordings, it manifested as a prominent depolarizing voltage sag in response to injection of hyperpolarizing current (Figure 2A–D). For several cells (*N* = 23), we made both voltage-clamp and current-clamp recordings. These generally yielded consistent data on the presence of *I*_h_ in a given cell: either both the slow hyperpolarization-triggered inward current and depolarizing sag were present, or both were absent. Two atypical cells exhibited a very small depolarizing sag when strongly hyperpolarized but no detectable slow inward current.

To characterize the behavior and pharmacology of the slowly developing current, cells were voltage clamped at ^−^73mV and subjected to a series of voltage steps from ^−^123 to ^−^83 mV in 10 mV increments (Figure 1 C–F). At voltage steps negative to ^−^83 mV, the instantaneous inward current response was followed by a slowly activating component noted above. That slowly activating component was generally small, with a mean amplitude of 35±18 pA (*N* = 97) measured at the steady-state of the response to the ^−^123 mV step. As expected for *I*_h_, it did not inactivate during the step (duration: 4–12s). Furthermore, it was blocked by extracellular application of the well-established *I*_h_ blockers Cs^+^ (3 mM; Figure 1C) or ZD7288 (100 µM; Figure 1E). The effects of Cs^+^ washed out several minutes after returning to control Ames' medium. The effects of ZD7288 did not wash out, in agreement with previous reports [26], [32].

In current clamp recordings, injections of hyperpolarizing current (^−^80 to ^−^10 pA; 500 ms) triggered a depolarizing voltage sag only during current injections that hyperpolarized the membrane beyond ^−^90 mV. The sag was virtually abolished by either ZD7288 (97±9% reduction in sag amplitude evoked by a ^−^60 pA current injection; *N* = 5; *P*<0.005; Figure 2A & B) or Cs^+^ (83±7% reduction in sag amplitude evoked by a ^−^60 pA current injection; *N* = 5; *P*<0.05; Figure 2C & D). These data are summarized in Figure 2E.

Additionally, both *I*_h_ blockers caused an increase in input resistance (R_N_) observable at hyperpolarized potentials. ZD7288 increased the R_N_ from 1004±178 MΩ to 1646±362 MΩ (64±27% increase; *N* = 7; *P*<0.001; Figure 2B) and Cs^+^ increased R_N_ from 777±144 MΩ to 1287±319 MΩ (64±16% increase; *N* = 5; *P*<0.05; Figure 2D). These data are summarized in Figure 2F. However, when R_N_ was measured near the holding potential (with ^−^10 pA current injections), it was unchanged by application of ZD7288 (*P* = 0.4; *N* = 7; compare square and triangle markers at the ^−^10 pA injection in Figure 2B) or Cs^+^ (*P* = 0.7; *N* = 5; compare square and triangle markers at the ^−^10 pA injection in Figure 2D). Thus, *I*_h_ appears to be inactive near the ipRGC resting potential.

**Figure 3:**
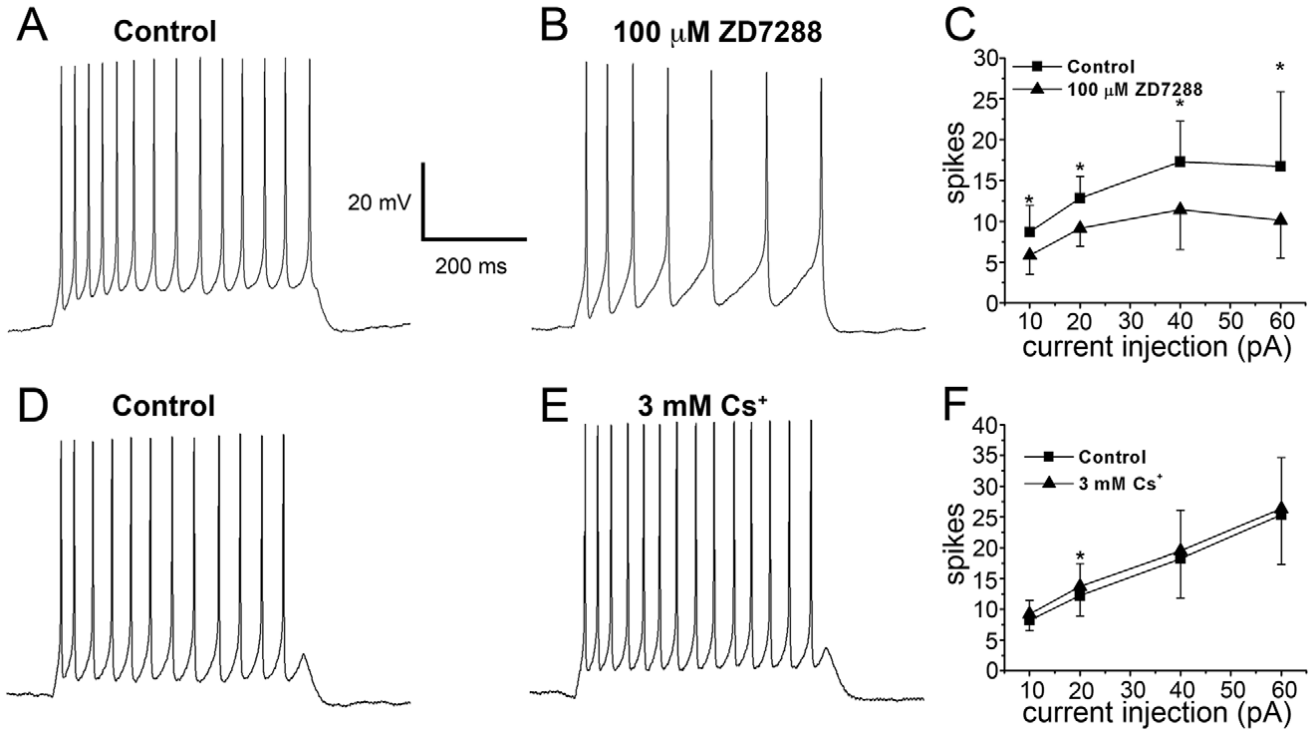
Differing effects of *I*_h_ blockers ZD7288 and Cs^+^ on spiking evoked by depolarizing current injections. **A**, **B**) Depolarizing current injections (20 pA) evoke trains of action potentials in an ipRGC. Spike frequency is lower in the presence of ZD7288 (**B**) relative to control (**A**). **C**) Group data for the effect shown in **A** and **B**. The difference is significant (* *P*<0.05) for all intensities of current injection (10–60 pA; *N* = 7 cells). **D**–**F**) Failure of the alternative *I*_h_ blocker Cs^+^ (3 mM) to cause a similar reduction in evoked action potentials. The treatment even caused a small increase in spiking for a 20 pA current injection (*N* = 4; * *P*<0.05). Vm was maintained near ^−^72 mV throughout for all cells (see Methods).

ZD7288 had an effect on the electrical behavior of ipRGCs that cannot be readily explained by its blockade of *I*_h_. It significantly suppressed the spiking evoked by depolarizing current injections (10–60 pA; *P*<0.05 for all current injections; *N* = 7; Figure 3A–C). This effect is presumably unrelated to blockade of *I*_h_ because extracellular Cs^+^, the alternative *I*_h_ blocker, had no significant suppressive effect on spiking (Figure 3D–F). There are precedents for suppressive actions of ZD7288 on other ion channels [33], [34].

*I*_h_ is also known to play a role in the rebound excitation at the termination of hyperpolarizing current injections [27], [35]. All recorded cells exhibited this characteristic rebound depolarization and, in many cases, rebound spiking as well. While blockade of *I*_h_ appeared to abolish this rebound depolarization in several cases, it did not do so in all. In several recordings, the rebound depolarization and spiking was diminished or abolished following application of ZD7288 (*N* = 4 cells) while in others it was enhanced or unchanged (*N* = 3 cells). Although Cs^+^ generally had no effect on the rebound (*N* = 4 cells), in one recording, rebound spiking was enhanced. The mixed effects of *I*_h_ blockers may be attributable to non-specific effects of ZD7288 on T-type Ca^2+^ currents [33], which also play a role in rebound depolarization [35]. Because there were frequently action potentials riding on top of these rebound depolarizations, we were unable to thoroughly assess the contribution made by *I*_h_.

### Ion selectivity

**Figure 4:**
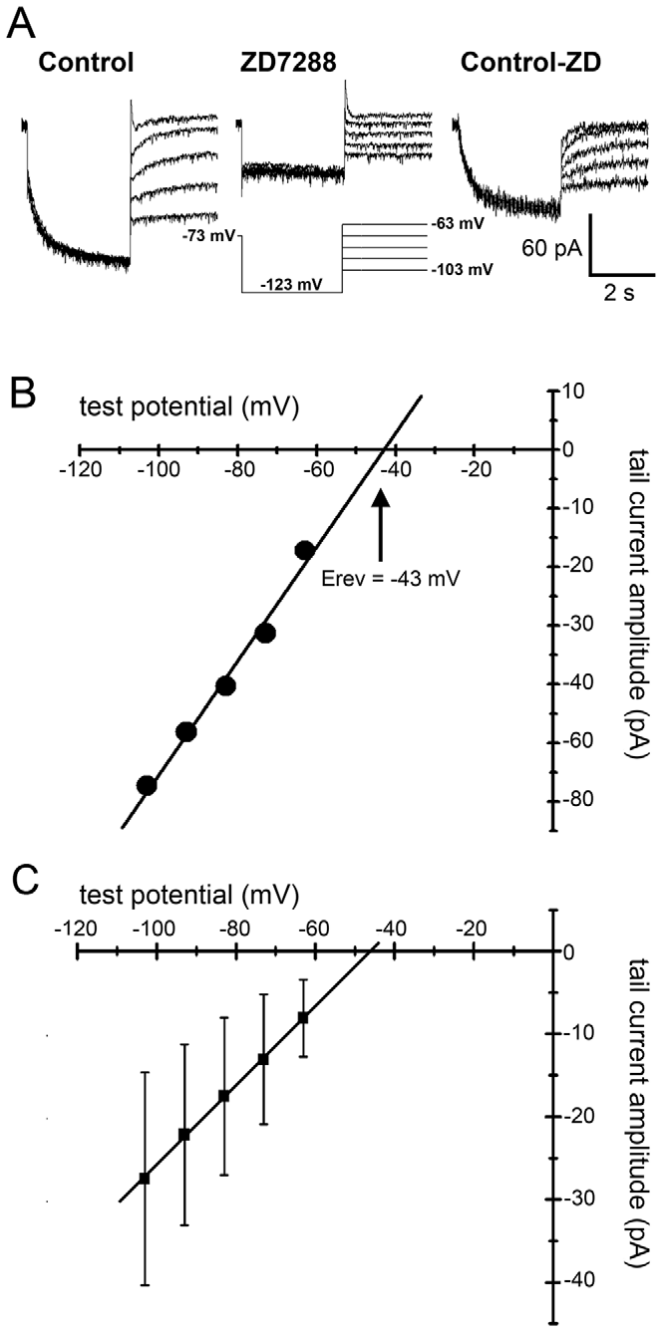
Current-voltage relationship of the fully activated *I*_h_ as measured by tail current analysis. **A**) *I*_h_ was activated by a step to ^−^123 mV and tail currents were recorded at a variety of test potentials ranging from ^−^103 to ^−^63 mV (left). This voltage protocol was repeated in the presence of the 100 µM ZD7288 (middle). The ZD7288 traces were subtracted from the control traces to isolate *I*_h_ (right) and these tail currents were fit with a single exponential to extrapolate the amplitude at the termination of the ^−^123 mV step. **B**) Tail current amplitudes as a function of test potential for the cell in **A**. A linear fit was used to extrapolate the reversal potential of *I*_h_, approximately ^−^43 mV for this cell. **C**) Tail current amplitudes as a function of test potential and the linear fit for all cells included in this analysis (*N* = 8).

*I*_h_ is carried primarily by monovalent cations with a slight preference for K^+^ over Na^+^. Although there is a small Ca^2+^ component (<1% of total current) [36], [37], it tends to be ignored in most analyses of ion permeability. To examine the reversal potential and ion selectivity of *I*_h_ in ipRGCs, we performed a tail-current analysis (Fig. 4). To obtain the *I–V* relationship of the fully activated current, the membrane potential was held at ^−^123 mV for 3–4s and then stepped to a series of test potentials. We repeated this voltage protocol in the presence of ZD7288 (100 µM) and subtracted the ZD7288 traces from the control traces (Figure 4A). Tail current amplitudes at each test potential were measured by fitting with a single exponential [25] and the reversal potential was calculated for each cell by linear extrapolation (Figure 4B & C). The mean reversal potential was ^−^43±11 mV (*N* = 8). From these values, we derive (as described in Methods) a relative Na∶K permeability ratio of 0.16±0.09 (E_Na_ = 89 mV; E_K_ = ^−^93 mV; 33°C). Thus, our data are consistent with *I*_h_ in ipRGCs being a mixed cation conductance with a moderate preference for K^+^ over Na^+^.

### Kinetics, activation, and I–V relationship

*I*_h_ may be carried by any of four HCN channels (HCN1-4). These differ from each other in their kinetics and half-maximal voltage of activation (V_1/2_). To infer which of these channels might mediate *I*_h_ in these neurons, we measured the time constants of activation (τ_act_) and deactivation (τ_deact_) of the current as well as its V_1/2_ of activation.

**Figure 5:**
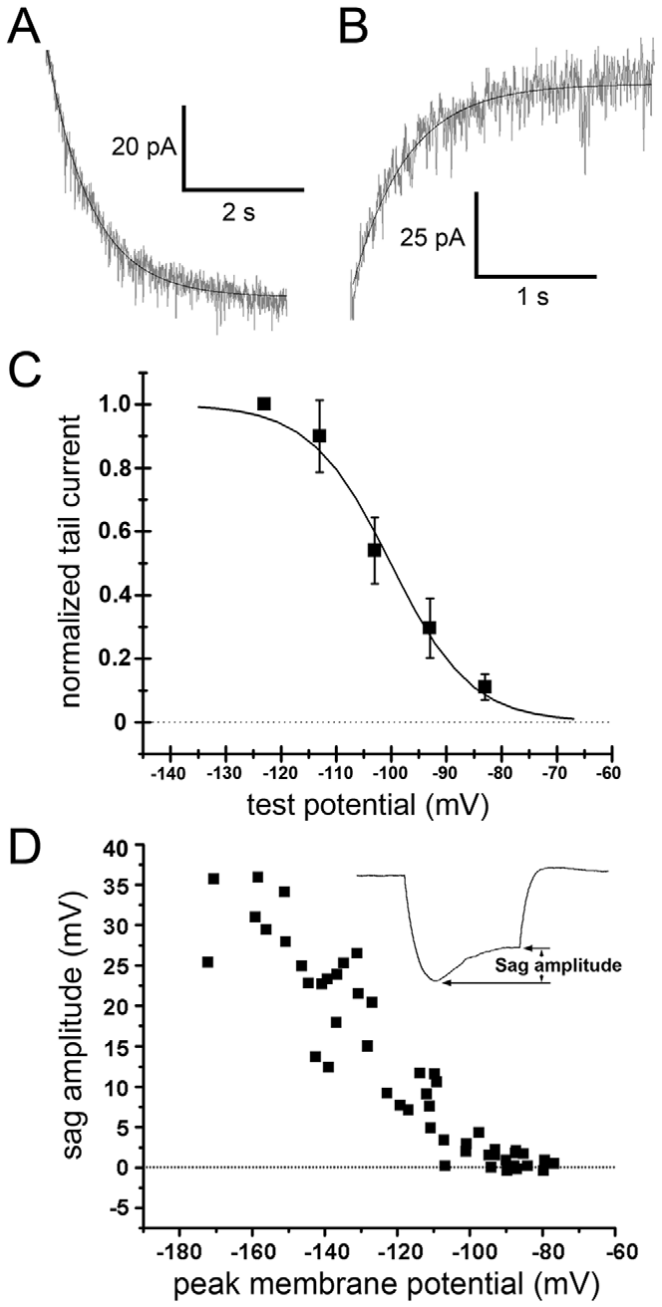
Activation and deactivation of *I*_h_. **A**) Activation of *I*_h_ in an example recording in response to a ^−^123 mV voltage step fit with a single exponential used to calculate the time constant of activation. **B**) Deactivation of *I*_h_ as estimated from a single exponential fit to the tail current from the same recording as in **A** after the membrane potential was stepped back to ^−^73 mV. **C**) The activation curve reflecting tail current amplitude measured at the end of steps to hyperpolarized test potentials and normalized to the tail current amplitude following a step to ^−^123 mV. **D**) Plot of sag amplitude (steady-state minus peak hyperpolarization, **inset**) from current-clamp recordings in which the membrane was hyperpolarized by current injections of ^−^80 to ^−^10 pA.

τ_act_ was measured by fitting a single exponential function to the slowly activating component of the current response to a voltage step to ^−^123 mV (Figure 5A). This yielded an average τ_act_ of 891±464 ms (*N* = 11). τ_deact_ was measured by fitting a single exponential to the tail current observed upon repolarizing the membrane to the holding potential (^−^73 mV) after stepping it to ^−^123 mV for 3–4 s (Figure 5B). This yielded an average τ_deact_ of 405±148 ms (*N* = 11).

Activation curves (Figure 5C) were constructed by fitting the ZD7288- or Cs^+^-sensitive tail current of individual cells with a Boltzmann function. The mean V_1/2_ was ^−^100±3 mV (*N* = 7) with a slope factor of 6.5±1.4 (*N* = 7). The *I*_h_ activation threshold was ^−^81.4±5.1 mV (*N* = 7). This threshold was confirmed in current clamp recordings; the sag did not appear unless the membrane was hyperpolarized beyond approximately ^−^80 mV (Figure 5D). Although the *I*_h_ activation curve reached a plateau at potentials negative to ^−^123 mV (Figure 5C), the sag amplitude continued to increase with increasingly hyperpolarized peak membrane potential (Figure 5D). This is presumably because *I*_h_ activation ultimately draws the membrane toward the equilibrium potential (approx. ^−^43 mV, Figure 4). Thus, the greater the divergence from that equilibrium potential at the peak of the voltage deflection, the greater the driving force for *I*_h_ ions and the greater the effect they exert on the cell's membrane potential.

The holding potential used in the foregoing voltage-clamp experiments was fairly close to the activation threshold, but this did not distort the activation curves. When we used, instead, a holding potential of ^−^53 mV, values of V_1/2_ (−101±3 mV, *N* = 4), slope factor (4.7±1.8, *N* = 4), and threshold (−87.2±5.8 mV, *N* = 4) were statistically indistinguishable from those obtained at ^−^73 mV (*P* values of 0.9, 0.1 and 0.2, respectively; independent Student's *t*-test; not shown). Furthermore, although *I*_h_ is known to be subject to rundown in whole-cell recordings and intracellular dialysis would have been rapid and pronounced in our recording configuration, we doubt that this contributed to the *I*_h_ activation range in our study. In several recordings made in the perforated patch configuration (*N* = 3; not shown), the activation parameters (V_1/2_ = −99.3±5.4; slope factor = 5.6±2.4; threshold = −82.8±2.8 mV) did not significantly differ from those obtained from whole cell recordings (*P* values of 0.7, 0.6, and 0.6, respectively; independent Student's *t*-test). Measures of the *I*_h_ activation range have also been shown to vary depending on the length of the hyperpolarizing step used to evoke *I*_h_ [38]. To assure that this did not interfere with our measurements, we made several recordings in which we used longer (10 to 12s) hyperpolarizing steps to activate *I*_h_ (1). Because cells tend to become unstable with extreme hyperpolarizations and because *I*_h_ reaches steady-state more quickly at more hyperpolarized potentials, we used shorter (4s) steps at ^−^113 and ^−^123 mV. Although V_1/2_ and threshold were slightly more positive when measured with long steps (^−^96.2±5.4 mV and ^−^75.4±10.0 mV, respectively; *N* = 4), these values did not significantly differ from those obtained with short pulses (*P* = 0.2 and *P* = 0.4, respectively; independent Student's *t*-test). Therefore, any error introduced by using 4-second steps rather than 10-second steps was very small, on the order of a few millivolts. In its slow activation kinetics and unusually negative V_1/2_ of activation, *I*_h_ in these cells resembles that carried by HCN4 channels [29], [39].

### Role of *I*_h_ in setting resting potential

**Figure 6:**
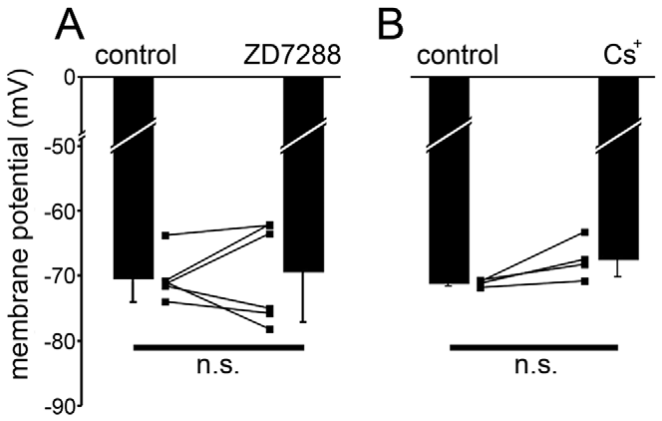
Blockade of *I*_h_ does not affect ipRGC membrane potential. Membrane potential was maintained near ^−^72 mV with DC injection before the addition of the pharmacological agent. Neither ZD7288 (**A**; 100 µM; *N* = 6) nor extracellular Cs^+^ (**B**; 3 mM; *N* = 4) had a significant effect on membrane potential. n.s. *P*>0.05.

In many cell classes, including retinal ganglion cells, *I*_h_ is an important contributor to resting membrane potential [23], [27], [40]. If *I*_h_ were active in ipRGCs at rest, the inward cation flux would provide a depolarizing influence, drawing the membrane potential toward the *I*_h_ reversal potential (^−^50 to ^−^20 mV) [27]. Therefore, we tested whether blocking *I*_h_ would hyperpolarize ipRGCs. Neither of the blockers of *I*_h_ (ZD7288, 100 µM, Figure 6A; Cs^+^, 3 mM, Figure 6B) caused a significant change in membrane potential (ZD7288: 0.94±6.4 mV depolarization; *N* = 6; *P*>0.05 and Cs^+^: 2.4±3.7 mV depolarization; *N* = 4; *P*>0.05). In these experiments, effective blockade of *I*_h_ was confirmed by suppression either of the depolarizing voltage sag in current-clamp recordings or of the slow inward current in voltage-clamp recordings, or both. This finding, which suggests that *I*_h_ is inactive at the resting membrane potential of these cells, is consistent with activation curves showing that the *I*_h_ activation threshold lies near ^−^80 mV (Fig 5B & C).

### Role of *I*_h_ in the ipRGC light response

**Figure 7:**
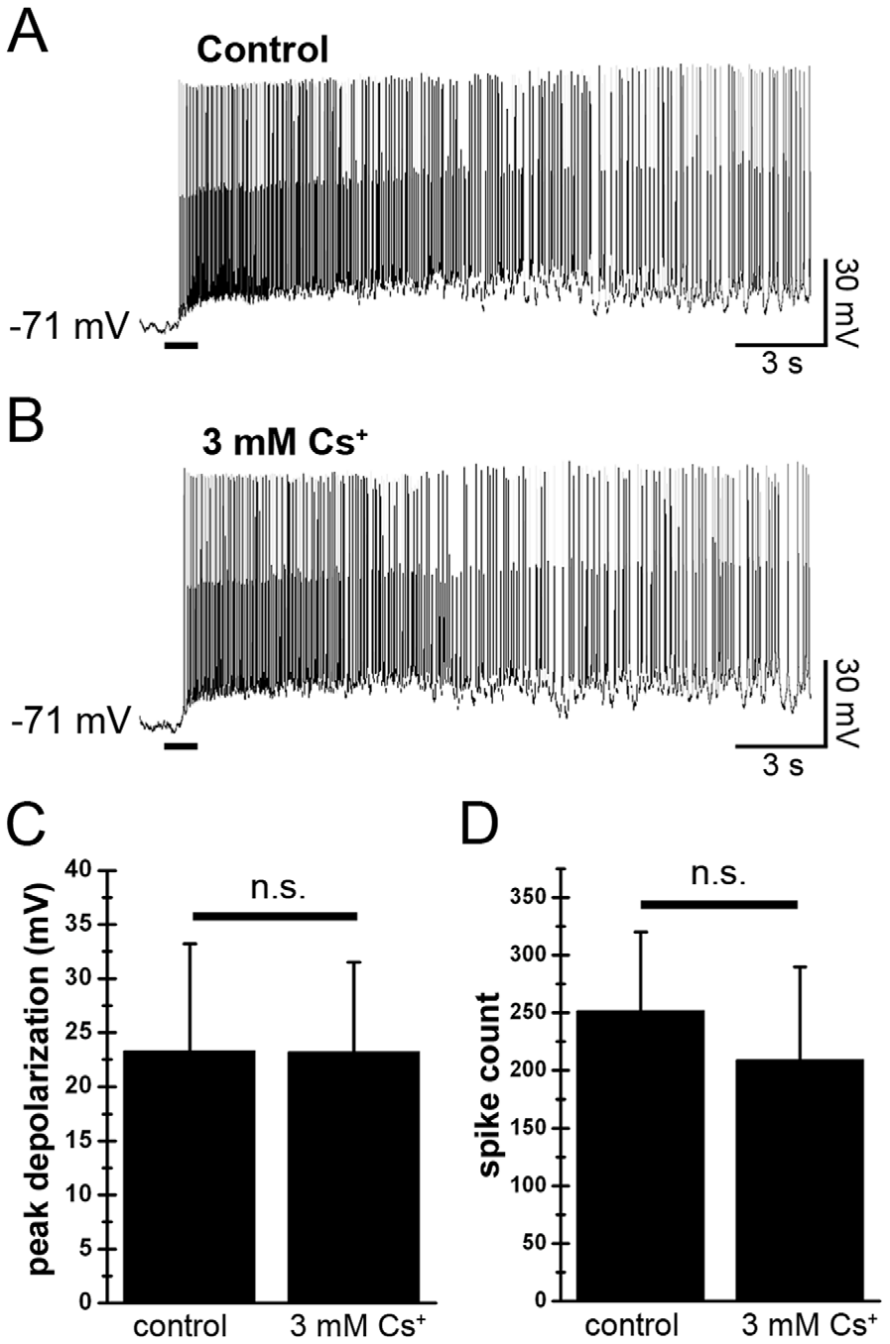
Blockade of *I*_h_ does not affect the ipRGC light response. Light responses were evoked by a 1 s flash of light (black bar; intensity = ^−^1 log *I*) before (**A**) and after (**B**) bath application of 3 mM CsCl. Blockade of *I*_h_ was confirmed by the loss of a depolarizing sag during 500 ms hyperpolarizing current injections. Cs^+^ had no effect on either the depolarization (**C**; *N* = 5) or the number of spikes (**D**; *N* = 3) evoked by the light flash. n.s. *P*>0.05.

Although our data indicate that *I*_h_ does not activate unless ipRGCs are substantially hyperpolarized, we tested whether it might play a role in their melanopsin-driven light responses. To do this we measured the light-evoked depolarization and spiking before and after blockade of *I*_h_ with Cs^+^ (Figure 7). Cs^+^ did not affect the light-evoked depolarization (23.3±9.9 mV in control; 23.2±8.3 mV in the presence of 3 mM Cs^+^; *N* = 5; *P* = 0.98). Cs^+^ also had no significant effect on the number of light-evoked action potentials (251±69 spikes in control conditions; 209±81 spikes in the presence of Cs^+^; *N* = 3; *P* = 0.13). Thus, *I*_h_ appears to play no role in the melanopsin-driven light response of ipRGCs.

## Discussion

### *I*_h_ in ganglion-cell photoreceptors

This study demonstrates the presence of the hyperpolarization-activated current *I*_h_ in intrinsically photosensitive retinal ganglion cells. The observed current shares the basic characteristics of *I*_h_ in other cell types, namely a slow, inwardly-rectifying current activated by membrane hyperpolarization. It is blocked by the bradycardic agent ZD7288 and by extracellular Cs^+^ at millimolar concentrations. An additional indicator of the presence of this current was the depolarizing voltage sag observed during hyperpolarizing current injection. This phenomenon, classically attributed to *I*_h_ [27], was also blocked by ZD7288 and Cs^+^. These phenomena have been described in previous studies of retinal ganglion cells in rats and other species [22]–[26]. Tail current analysis was also consistent with descriptions of *I*_h_ in a host of other systems. As elsewhere, *I*_h_ in ipRGCs reverses near ^−^40 mV and is a mixed cation current with a moderate preference for K^+^ over Na^+^ [27]. It should be noted that the ipRGCs from which we recorded were retrolabeled from the SCN and are thus mainly or exclusively of the M1 subtype [7]. Therefore, the conclusions of this study are applicable to M1 cells rather than all ipRGCs.

Heterologous expression studies in oocytes and HEK293 cells have shown that *I*_h_ is carried by four varieties of HCN channels, designated HCN1-4 [28], [29], [39], [41]–[43]. The four HCN channels differ from each other most notably in their activation kinetics and voltage of half-maximal activation (V_1/2_). In rat ipRGCs, our data indicate that *I*_h_ has an activation time constant near 900 ms for a voltage step to ^−^123 mV and a V_1/2_ of approximately ^−^100 mV. Both of these measures are consistent with *I*_h_ carried by HCN4 channels, which tend to activate more slowly and have a slightly more negative V_1/2_ than currents carried by HCN1-3 [29].

The inference that rat ipRGCs probably express HCN4 channels is consistent with available immunohistochemical data. Oi et al. [44] demonstrated HCN4-like immunoreactivity in many ganglion cells of the rat retina and Müller et al. [45] noted in passing that some cells in the ganglion cell layer of rat retina were HCN4 immunopositive (but see also [46]). That said, we cannot exclude the possibility that ipRGCs express more than one HCN channel protein that forms functional heteromers with HCN4. Also, our recordings were performed in isolated cells that were entirely devoid of dendritic processes. Therefore, the activation parameters we report apply only to *I*_h_ in the soma. It may be that a different set of HCN isoforms expressed in the dendrites would confer a different activation range on *I*_h_ in intact ipRGCs. Additionally, because HCN channel isoforms are differentially regulated by various intracellular messengers [47], they may exhibit different sensitivity to damage or disruption of regulatory processes during the dissociation procedure.

### *I*_h_ in rat ipRGCs does not set the resting membrane potential

Perhaps best known for its role in cardiac pacemaking, *I*_h_ has also been implicated in neuronal function [27], including synaptic integration [48]–[51], rhythmic oscillation [52], [53], and rebound depolarization [35]. In some neurons, including some RGCs, *I*_h_ is tonically active at rest [23], [25], [26], [40], [54]. This drives the resting membrane potential towards the *I*_h_ reversal potential of ^−^50 to ^−^20 mV; thus, blockade of *I*_h_ triggers membrane hyperpolarization. In ipRGCs, however, neither of the *I*_h_ blockers tested affected the membrane potential, suggesting that *I*_h_ is not active in ipRGCs at rest. This is corroborated by our finding that blockade of *I*_h_ does not affect the light response and that the membrane must be hyperpolarized beyond about ^−^80 mV to activate *I*_h_ in these cells. This is far more hyperpolarized than the reported resting potential of ipRGCs, approximately ^−^60 mV in perforated patch recordings [14]. Similar values of resting potential (approx. ^−^45 to ^−^75 mV) have been reported in conventional patch recordings of these cells [1], [8], [20], [30], [55], [56] although such data could be distorted by intracellular dialysis. Since *I*_h_ is not active at rest, ipRGCs apparently differ from other rat RGCs [25], [26]. We performed several control experiments, measuring *I*_h_ activation curves with longer pulses, a more depolarized holding potential or in perforated patch configuration and none of the activation curves differed substantially from that illustrated in figure 4C. Perhaps these cell groups differ in HCN channel type, splice isoform or regulatory state [39], [47].

### Non-specific effects of ZD7288

In the course these studies, we found that the widely used *I*_h_ blocker ZD7288 moderately suppressed evoked spiking. Because an alternative *I*_h_ blocker, extracellular Cs^+^, did not replicate this effect on spiking, these effects of ZD7288 are independent of its blockade of *I*_h_. Indeed, ZD7288 has recently been shown to inhibit T-type Ca^2+^ currents [33] and K^+^ currents [34]. Furthermore, *I*_h_ blockers had inconsistent effects on rebound depolarization that may have resulted from non-specific actions of ZD7288 on T-type Ca^2+^ currents. Thus, while ZD7288 is widely considered a specific blocker of *I*_h_ and is commonly employed in studies seeking to examine its contributions to neuronal function [57], our results and those of other groups [33], [34] warrant cautious interpretation of such studies. Additionally, this raises the possibility that T-type Ca^2+^ channels contribute to the spiking behavior of ipRGCs.

### Functional relevance of *I*_h_ in ipRGCs

The functional relevance of *I*_h_ in this particular class of RGCs is unclear. In classical photoreceptors, *I*_h_ shapes the temporal characteristics of the light response, drawing the membrane potential towards the depolarized dark state during hyperpolarization induced by long and intense photic stimulation [58]–[63]. *I*_h_ may similarly constrain the hyperpolarization evoked by light stimulation of the receptive-field center of OFF ganglion cells or the OFF surrounds of ON-center ganglion cells. It may also enhance rebound depolarization and spiking when such light stimuli terminate [23].

In ipRGCs, however, both the intrinsic and synaptically-driven light responses are exclusively depolarizing under physiological conditions and therefore do not bring the ipRGC membrane potential near the *I*_h_ activation threshold [1], [20]. While ipRGCs are subject to ionotropic GABA and glycine-gated inhibitory currents [19], [20], these probably do not hyperpolarize the membrane sufficiently to activate *I*_h_ because the Cl^−^ reversal potential presumably lies close to the resting membrane potential and well positive to the *I*_h_ activation threshold. Thus, the activation range for *I*_h_ may lie well outside of the normal operating range of this class of ganglion cells.

This conclusion may be premature, however, because dynamic modulatory influences could substantially alter the properties of *I*_h_ described here. Of particular interest in this regard is evidence that the HCN channels that carry *I*_h_ are subject to modulation by cyclic nucleotides [27]. This is especially true of HCN4, which our data suggest is the most likely substrate of *I*_h_ in these cells, and of HCN2 [28], [41], [42], [47], [64]–[66].

Dopaminergic innervation may provide a basis for such modulation in ipRGCs. The dendrites of M1 cells, which comprise the majority of cells from which we recorded, tightly costratify with the dopamine-releasing processes of dopaminergic amacrine cells in the outermost sublamina of the inner plexiform layer [67], [68]. Furthermore, we have preliminary evidence that ipRGCs express a D1-family receptor (M.J. Van Hook & D.M. Berson, *Association for Research in Vision & Ophthalmology*, 2009; but see [69]). Activation of D1 receptors should increase intracellular levels of cAMP [70] which could shift the activation curve of *I*_h_ so that its threshold lies within a physiologically relevant voltage range. Indeed, stimulation of D1 receptors is known to shift the *I*_h_ activation curve in some rat retinal ganglion cells by a cAMP-dependent mechanism, thereby reducing their input resistance and excitability [25]. Retinal dopamine levels are modulated by lighting conditions and circadian phase. Thus, dopaminergic influences on *I*_h_ could provide a basis for light adaptation and circadian modulation of ipRGCs and of the non-image-forming visual mechanisms they support [30], [71], [72].

*I*_h_ is also well known to play a role in synaptic integration at dendrites [48]–[51]. Our study was not suited to explore such a contribution in ipRGCs because their dendrites were lost during cellular dissociation. Thus, while ipRGCs clearly express *I*_h_, its role in ipRGC function and, by extension, non-image-forming vision remains to be elucidated.

## Supporting Information

Figure S1 ***I*** **_h_ activation with long hyperpolarized voltage steps.** *I*_h_ was evoked with hyperpolarized voltage steps from a holding potential of ^−^73 mV and the current in the presence of Cs^+^ was subtracted from the control condition to obtain *I*_h_ alone. 10 s long steps (**A**) were used for test potentials from ^−^103 to ^−^83 mV to allow *I*_h_ to reach steady-state, while, 4 s long steps were used to activate *I*_h_ at ^−^123 and ^−^113 mV (**B**). **C**) The activation curve constructed from tail currents measured at the point of repolarization to ^−^73 mV using a 10 s step (points and solid line; *N* = 4). Dotted curve: the activation curve obtained with 4 s steps (from Figure 5C), included for comparison.
(TIF)
