# Supplementary figures and images for: Hyperpolarization-Activated Current (I h) in Ganglion-Cell Photoreceptors
Source: PLoS One. 2010 Dec 20;5(12):e15344. doi: 10.1371/journal.pone.0015344 (PMC3004865; doi:10.1371/journal.pone.0015344)

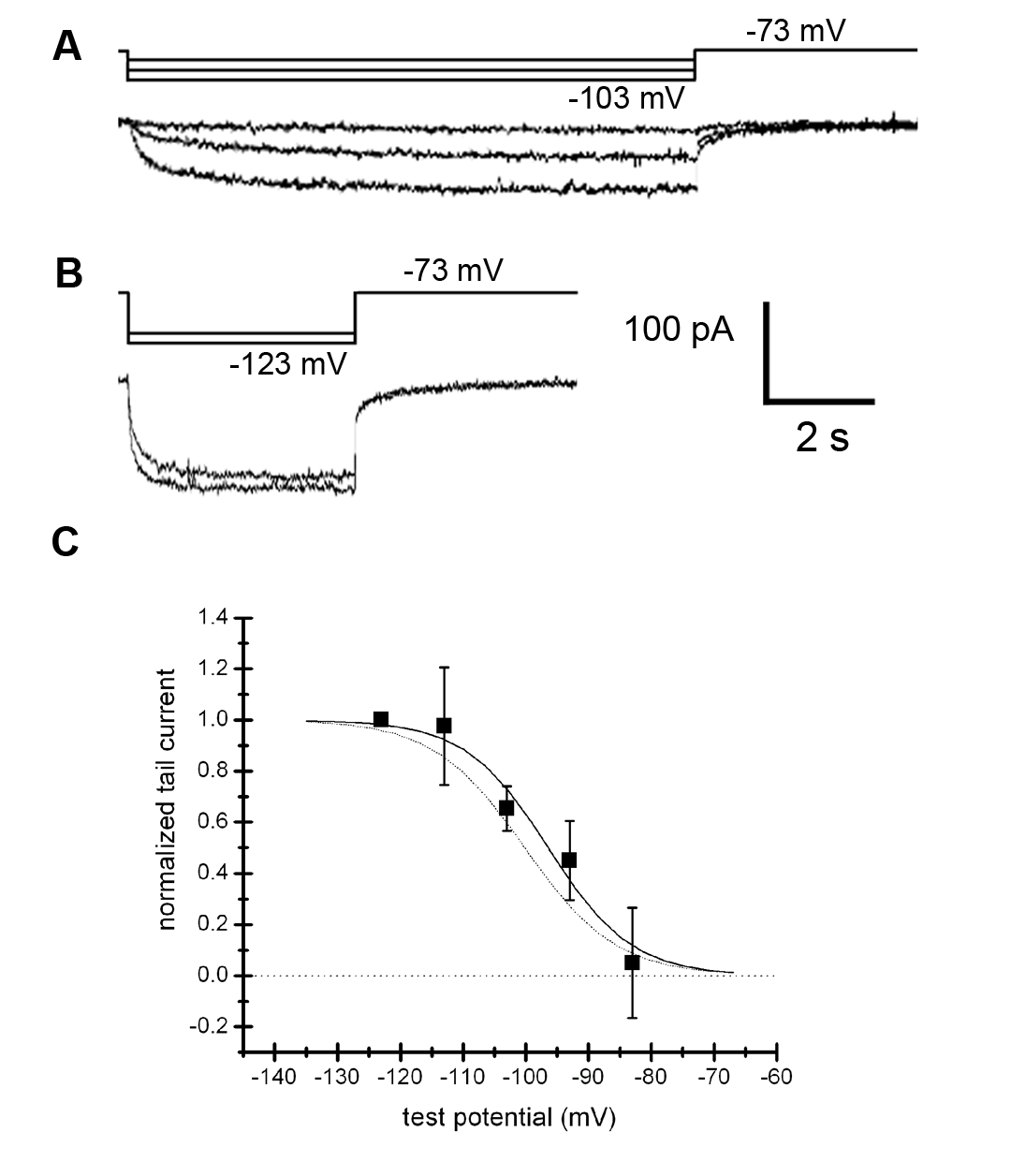

Supplement: Figure S1 — I h activation with long hyperpolarized voltage steps. I h was evoked with hyperpolarized voltage steps from a holding potential of −73 mV and the current in the presence of Cs+ was subtracted from the control condition to obtain I h alone. 10 s long steps (A) were used for test potentials from −103 to −83 mV to allow I h to reach steady-state, while, 4 s long steps were used to activate I h at −123 and −113 mV (B). C) The activation curve constructed from tail currents measured at the point of repolarization to −73 mV using a 10 s step (points and solid line; N = 4). Dotted curve: the activation curve obtained with 4 s steps (from Figure 5C), included for comparison. (TIF) [file pone.0015344.s001.tif]
